# A qualitative analysis of facilitators and barriers to physical activity among patients with moderate mental disorders

**DOI:** 10.1007/s10389-022-01720-4

**Published:** 2022-06-01

**Authors:** Denise van Rijen, Gill A. ten Hoor

**Affiliations:** Psychology Neurosciences Department, Maastricht University, PO BOX 616, 6229 ER Maastricht, The Netherlands

**Keywords:** Mental disorders, Determinants, Behaviour change theories, Physical activity, Barriers, Facilitators

## Abstract

**Aim:**

The current study aims to qualitatively identify determinants, barriers and facilitators of physical activity among a population with mental health disorders.

**Subject and methods:**

Seventeen participants with moderate mental disorders were recruited. Semi-structured interviews were conducted to identify physical activity facilitators and barriers. Data were organized and analysed in ATLAS.ti, mainly based on a generic qualitative research approach.

**Results:**

Most participants found physical activity important and expressed a positive attitude towards it. In general, higher self-efficacy and more social support were beneficial for participants’ physical activity levels. Reasons/facilitating factors to be more physically active were: having fun, good weather, progress, routine, self-compassion and a stimulating environment. Barriers were not having fun, being busy, mental complaints, lack of energy, procrastination and physical complaints.

**Conclusion:**

Future interventions could promote physical activity among people with moderate mental disorders to help them identify and overcome barriers. The newly identified determinant ‘self-compassion’ could be an interesting target for promoting physical activity in this group of people with moderate mental disorders.

## Introduction

Physical activity is important for our physical and mental health (World Health Organisation 2020). We define physical activity as any movement of the body (World Health Organisation) that includes leisure, household, occupational and transport movements (Cerin et al. 2009). However, 27.5% of the global population has an insufficient physical activity level (Guthold et al. 2018). In the Netherlands, there is a small positive trend in adherence to physical activity guidelines from 39.9% in 2001 to 46% in 2018 (Duijvestijn et al. 2020). However, still more than half of the Dutch population does not exercise enough according to the official guidelines (Duijvestijn et al. 2020). In particular, people with moderate or severe mental disorders have insufficient physical activity levels (Pelletier et al. 2017; Schuch et al. 2017; Vancampfort et al. 2017). However, little is known about why people with mental health disorders have more difficulties with initiating and maintaining physical activity (Schuch et al. 2017). Physical inactivity and its consequences can be called a pandemic that needs action (Kohl 3rd et al. 2012). Therefore, it is imperative to explore what determinants can help to increase physical activity among people with moderate mental disorders.

Insufficient physical activity increases the chance of non-communicable diseases and death. However, physical inactivity also affects various domains of mental health, such as depression, anxiety, and stress (Bélair et al. 2018; Czosnek et al. 2019; Gerber et al. 2018; Hiles et al. 2017; Pickett et al. 2012; Rebar and Taylor 2017; Rosenbaum et al. 2014; Schuch et al. 2019). Cerin et al. (2009) examined the relationship between leisure, household, occupational, and transport physical activity and mental health in healthy people. They found that only leisure-time physical activity had a consistently positive relationship with mental well-being; especially for people under the age of 57, and when they spent average or below-average time on household tasks. In this study, people spent the least time on leisure-time physical activity (Cerin et al. 2009). In sum, people with mental disorders are less physically active (Schuch et al. 2017) and subsequently, less physical activity influences their mental health negatively (Czosnek et al. 2019), which can lead to a negative vicious cycle.

### Barriers and facilitators of physical activity among people with mental disorders

To break the vicious cycle among people with mental disorders, it is essential to identify barriers and facilitators as potential intervention targets. A review of physical activity among people diagnosed with depression found that low self-esteem and higher levels of depressive symptoms were associated with lower physical activity (Vancampfort et al. 2015). A lack of knowledge of the importance, fear, negative experiences, and a lack of energy were associated with lower physical activities (Vancampfort et al. 2015). Among people with depression and other severe mental disorders, other common barriers that were found are mood (e.g. sad, angry), too shy/embarrassed to exercise, lack of enjoyment, belief that physical activity is boring, fear of social interaction, unsure what to do, feeling too old, considering themselves not as a sporty person, fear of being injured, lack of motivation, lack of time, costs, lack of environmental access, the weather, feel unsafe to go outside, lack of equipment and lack of encouragement/support from others (Biddle and Mutrie 2007; Firth et al. 2016; Glowacki et al. 2017).

People that suffer from anxiety disorders have, next to the previously discussed barriers, also anxiety specific barriers to performing physical activity. They frequently experience exercise anxiety and avoid physical activity to reduce the anxiety short term. In addition, they experience social evaluation barriers such as thoughts of being judged by others while they are exercising; negative evaluations from others about their exercise techniques or being judged by others for their physical appearance (e.g. sweating) (Mason et al. 2019). The misattribution of physiological effects is also a barrier people encounter with anxiety disorders. When they realize that exercising can have physiological sensations that are similar to sensations from anxiety, they start to avoid physical activity (Mason et al. 2019). Mason et al. (2019) showed that people with anxiety-related disorders have specific barriers to becoming more physically active and that they might need an alternative approach to increase their physical activity.

Factors that helped people to become more active are: returning to a more active self; maintaining their weight and health; managing their stress and pain; improving emotional wellbeing, flexibility, sleep, space to think, appearance, body image, overall mood, energy levels; and to build up their strength (Firth et al. 2016; Glowacki et al. 2017). Specific for people with depression, an increase in physical activity self-efficacy turns into an increase in positive affect which subsequently decreases levels of depression (Pickett et al. 2012). Physical activity can start from extrinsic motivation. However, when people experience the positive effects, it can turn into a more intrinsic motivation (Pickett et al. 2017). In summary, across people with mental disorders there are different barriers and facilitators, and which to target to promote physical activity depends on the mental disorder.

### Determinants of physical activity among people with mental disorders

Behaviour change theories are important to understand why and how physical activity might change (Michie et al. 2008). Behaviour change theories describe determinants to target in interventions, and therefore it is important to find out what specific determinants are among people with moderate mental disorders to increase their physical activity level. Zechner and Gill (2016) tested the social cognitive model among people with severe mental disorders. The Social Cognitive Model includes self-efficacy (belief and understanding of your capabilities) and outcome expectations (estimation of what the outcome will be of a specific behaviour) (Bandura 2001). The model states that when someone has a higher self-efficacy for exercise and a greater expectation of the outcome, they are more likely to use self-regulatory strategies to change their current state of physical activity. Zechner and Gill (2016) added social support from family and friends; barriers to exercise and goal-setting practices to Social Cognitive Model from Bandura (2001). They found that goal setting was the strongest predictor of physical activity. The relationship between self-efficacy and physical activity was mediated by goal-setting. In addition, the relationship between self-efficacy and goal-setting was mediated by outcome expectations. In other words, when someone has a positive idea about the outcome of physical activity, they are more likely to feel confident about performing the physical activity. The more confident someone feels, the better the chances are the person will make a plan to perform the physical activity. In conclusion, someone has a positive outcome in mind, feels confident and has a plan and this leads to more physical activity (Zechner and Gill 2016).

Motivation is another important aspect to perform physical activity (Biddle and Mutrie 2007). The Self-Determination Theory (Deci et al. 1994) distinguishes between autonomous (individuals integrate the activity into their sense of self) and controlled motivation (behaviour is performed because of external contingencies). The Self-Determination Theory explains that autonomous motivation can be increased by enhancing the need for autonomy, competence and relatedness (Deci et al. 1994). Farholm et al. (2017) tested the self-determination theory among people with severe mental disorders that were physically active and found that the need for autonomy, competence and relatedness had a positive effect on need satisfaction and subsequently a positive effect on autonomous motivation, but a negative effect on controlled motivation. Autonomous motivation increased their physical activity level and their physical health-related quality of life (Farholm et al. 2017).

### Current study

There are only a few studies that investigated the needs among people with moderate mental disorders that can be used for the development of (online) interventions to promote physical activity. The qualitative studies that exist investigate determinants among a specific group or physical activity such as alcohol and drug users (Horrell et al. 2020), anxiety-related disorders (Mason et al. 2019), football (Friedrich and Mason 2018) and rugby (Wilcock et al. 2021). The current study aims to qualitatively identify determinants, barriers and facilitators of physical activity among a broader population with mental health disorders among a broader definition of physical activity. This information will be used for the development of an online intervention to increase physical activity among people with moderate mental disorders. In addition, we identify their current physical activity level, how satisfied they are with their current physical activity level and their definitions.

## Method

### Research design and participants

The COREQ checklist is used to report this qualitative research explicit and comprehensive (see Appendix 1 for the full checklist; (Tong et al. 2007). Inclusion criteria were Dutch-speaking people registered with the mental health institution ‘Oh My Mood’ (https://www.ohmymood.com). Inclusion diagnoses were mood- and/or anxiety-related disorders; somatic symptom and related disorders; attention deficit and hyperactivity disorder (ADHD); and general complaints (e.g. emotional problem, ruminating, perfectionism, insecurities). Exclusion criteria were personality disorders, psychosis, addictions, high risk of suicide, and complex trauma. They were excluded because they need more comprehensive treatment than Oh My Mood can offer, where the study was conducted. We aimed to recruit participants that were minimally exposed to treatment, so their physical activity level was minimally influenced and results are generalizable; 24 potential participants were contacted for an interview, of whom four did not answer the telephone, 17 agreed on participating and three were not interested in the topic or found treatment already intense. Recruitment stopped when data saturation was reached and participants were providing similar responses (Moser and Korstjens 2018). Participants did not receive any incentive for the study.

### Procedure

After people had their first contact with the mental health institution, they were contacted by the first author who was also an employee of the Mental Health institution. Only people that already gave consent for anonymous use of data in their treatment agreement were contacted about whether they were willing to participate in an interview about physical activity. During the invitation, people were informed about the study and gave their consent, after which an interview was scheduled. At that point, people did not have a diagnosis yet; however, by the time the interview took place they had an official diagnosis. All people met the inclusion criteria. Because of the COVID-19 situation, all interviews took place over a video call using the online platform ‘Whereby’ (https://whereby.com/), so people could participate from home, only with the interviewer and participant present. After the interview was over, people could stay for a debriefing and ask questions. Participation in the interview did not affect potential treatment within Oh My Mood in any way. During the procedure, the therapist was always available for the interviewer and the client. Ethical approval was obtained from the Ethics Review Committee Psychology and Neuroscience (ERCPN-1880_10_02_2018).

### Interviews

Before the interview started participants were asked for their consent to participate in the interview, to use their answers anonymously and to audiotape the conversation to analyse their answers (see appendix 2). The 17 interviews took place in May 2021 and were conducted, recorded and transcribed by the first author, who invited the participants. A semi-structured interview is used with a general list of descriptive questions. The questions were not pilot tested but were adjusted during the interviews. Because of the nature of the interview, it depended on the participants’ answers to what was asked next. Interview questions were based on the Social Cognitive Model (Zechner and Gill 2016), the Self-Determination Theory (Deci et al. 1994; Ryan and Deci 2000) as explained in the introduction and the Theory of planned behaviour (Ajzen 1991). The Theory of Planned Behaviour states that someone’s attitudes, subjective norms and perceived behavioural control lead to an intention and subsequently increase a health behaviour (Ajzen 1991; see Appendix 3 for the outline and examples). The first 12 interviews were visually taped with only the interviewer visible. The other five interviews were audiotaped, so the participants were visible to the interviewer during the conversation. The interviews were approximately 30 minutes long.

### Data analysis

**Fig. 1:**
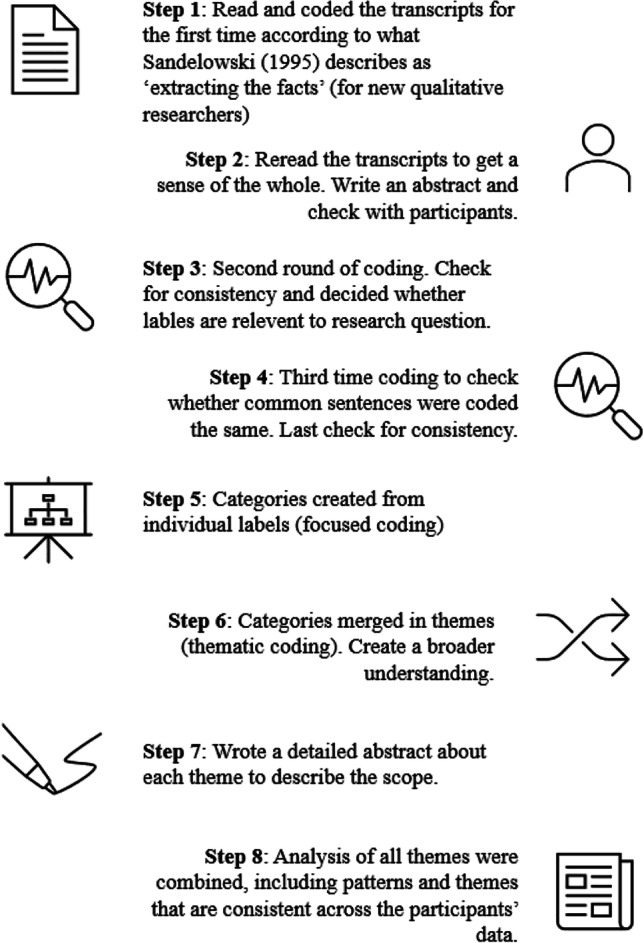
Eight-step process of qualitative analysis. Note. Step one is based on the work of Sandelowski (1995). The steps are based on the inductive analysis approach described by Thomas (2006) and Percy et al. (2015)

Data analysis was based on a generic qualitative approach. After the interviews took place, the interviews were transcribed. The first author reviewed all transcripts in ATLAT.ti version 9.0 (ATLAS.ti Scientific Software Development GMBH, Berlin, Germany) and an eight-step process was used for analysing the data (see Fig. 1 for an overview of the different steps). Several strategies were applied to improve the reliability and validity of the data. Transcripts were first coded within a week after they were conducted. The transcripts were reread after coding and a full abstract was made to return to the participants for comments and approval (member check: (Korstjens and Moser 2018). One participant asked for a different interpretation of a goal. After the approval of the participants, the second round of coding took place. After the second round of coding, all transcripts were reread in one day to adjust the codes if needed and to check whether codes were used the same across all transcripts (method triangulation: (Korstjens and Moser 2018). In addition, one expert analysed the transcripts to confirm the credibility of the data (investigator triangulation: (Korstjens and Moser 2018). Lastly, the interviewer reflected on how her assumptions, preconceptions and values could have influenced the answers (reflexivity: (Korstjens and Moser 2018).

## Results

### Descriptives

Seventeen participants finished the interview (64.71% female, mean age 31.94, range 19–57). The participants had the following diagnosis: Depression (in any form except for a major depressive disorder), specific anxiety disorder, general anxiety disorder, attention deficit hyperactivity disorder, insomnia, post-traumatic stress disorder, panic disorder, obsessive-compulsive disorder and somatic symptom disorder. Participants’ definitions of physical activity can be interpreted on a continuum, ranging from ‘physical activity is everything that is not sitting or sleeping’ to ‘more moderate/vigorous exercises in combination with feelings of being satisfied’. See Appendix 4 for all given definitions of physical activity. The weekly physical activity levels of participants were divergent. The least active participant only conducted some household tasks. The most active participant did some light exercises after waking up, walked every day for at least 30 minutes and performed moderate to vigorous physical activity approximately three times a week. Participants can be distinguished into four groups: not active at all (1), active during only daily physical activities (1), active during only moderate/vigorous physical activity (3) and active during both (4). Almost all participants expressed the intention to increase their physical activity level, except for the four people that were in the ‘active during both’ group.

No consistent pattern was found in satisfaction level and actual physical activity level. The mental/physical circumstances of the person, negative thoughts, high demands on themselves and self-compassion seemed to play a crucial role in the satisfaction level. All participants, except for one, found physical activity important. The most important reason for participants to perform physical activity were physical reasons such as increasing aerobic fitness, increasing posture, healing past injuries, improving strength, losing weight and increasing physical appearance. The second important reason was to improve their mental health (e.g. increasing mood, feeling better about themselves, clearing the mind and increasing well-being).

### Determinants of physical activity

#### Attitudes

The majority of the participants expressed a positive attitude towards physical activity. However, some of them experienced a negative attitude, especially against more moderate/vigorous physical activity. Often a threshold to start with physical activity is described, but when they started/overcame this threshold, they enjoyed it. One participant explained that she experiences a threshold, but sometimes she comes into a flow of performing physical activities. *‘Sometimes I have periods when I am in such a flow of doing a lot of physical activities, eh, yes then I really do feel better. Kind of an addictive feeling’* (P6). Explanations given for the positive attitude are: putting your energy into something, stress relief, taking your mind off it, giving your daily life meaning, feeling proud of yourself, and feeling happier afterwards. Most explanations are related to their mental health such as this participant explains: *‘Physical activity is progress on all aspects of life, you stay clear, fresh, it has a positive influence on your body and your mind, it helps to be more present’* (P2).

#### Self-efficacy

More than half of the participants talked about difficulties to start with physical activities. Different given reasons are *‘Because I am lazy’* (P5, P10*)*, *‘I lack self-discipline’* (P3, P5, P7, P17), and *‘I am not motivated enough’* (P3, P7, P8, P9, P10, P13, P15), *‘I am not a sporty type’* (P8, P9, P15), or a lack of confidence if they can perform the actual movement. Participants in the active during both groups showed more confidence than people that are not active at all or performed more daily movements. Participants who performed only moderate/vigorous physical activity experienced a low self-efficacy before they became more active.

#### Social support and norm

Almost all participants experienced enough social support to perform the physical activity. Their social network did not counteract their intention to become (more) physical active. Two participants did not experience enough social support. These participants showed the lowest physical activity level. In general, participants enjoyed physical activity more when done with other people. Good guidance from a personal trainer or someone who is experienced is beneficial for the participants. Compliments from their surrounding are perceived as motivating. So is described: *‘Even if it is a compliment or a “Hey you look good” or whatever, that is motivating, it shows that you are doing well’* (P4). Other people can function as a foot-in-the-door to commit to their intention to perform physical activity. An example is, *‘Then umh, there is someone, yes, a kind of social control. Someone is waiting for you, so it is easier once you have said “I am in to go.” Then you have to go. So that makes it easier for me’* (P9). A strong social norm in combination with having great demands on themselves seemed to be counterproductive for their physical activity level. So mentioned one participant: *‘Umh, but then I sometimes have the thought: “my mother is 60 and she can run 10 kilometres in an hour, so why can I not do it?” Because I basically have the same posture. So, I should be able to do so let’s say’* (P9). In general, a higher social norm resulted in performing more physical activity.

#### Facilitators and barriers to physical activity

In most interviews, the weather is discussed as a facilitator/barrier. More than half of the participants said their physical activity depended on the weather. However, other facilitators or barriers were more prominent. Appendix 5 shows a full overview of facilitators, barriers and tricks that helped participants to become more physically active. The most frequently mentioned facilitators were social support; having fun during physical activity; noticing progress; performing physical activity in a daily routine; self-compassion; and being in a nice environment. The most frequently mentioned barriers were being busy with other appointments; mental complaints; lack of energy; negative thoughts; not having fun during physical activity; procrastinating; and physical complaints/(fear of) injuries.

#### Social support/buddy/commitment

All participants found it easier to become more active when they experienced enough social support (such as having a physical activity buddy) or when they felt committed to something. One participant described: *‘So for myself, I can leave it very easily, but if I let someone else down, I will find it more difficult’* (P15). Another participant said: ‘*But if I have an appointment with Umh, a friend, then I force myself to go outside, to honour the deal*’ (P3). Only a few participants did not experience enough social support. Some of the participants mentioned some reasons why they felt empowered to perform more physical activity after they encountered social support. One participant described that she felt upset when she saw her partner doing it better. However, she came out stronger after they talked about it and felt more supported. ‘*But we do learn to talk about it. Umh, we explained to each other our struggles. And because of that, you can also embrace better what you already have*’ (P2).

#### (Not) Having fun during physical activity

Having fun during physical activity was mentioned, across all groups of physical activity levels. Some participants explored different physical activities before they found what they really liked. One participant explained: *‘If you want to become active, find something that you like. Umh, because when you do not like it, your motivation is hard to find. And if you have something you really like, yes then you are also more motivated. You can keep doing it, otherwise, you will be tired of it after a week or a month’* (P12).

#### Being in a nice environment and noticing progress

To become more active, it helped people to be outside or in a nice environment. Some physical activity helped them to explore their neighbourhood/new places. For example, this participant explained: ‘*Because it helps me to see and do all kinds of fun things of course. Umh, yes, it helps me to be able to go somewhere else*’ (P11). After people started a physical activity, they became more motivated to continue when they noticed some progress. *‘That you get better at something, because then, of course, it becomes more and more fun to do’* (P14).

#### Physical activity is part of daily routine/busy with other appointments

One of the most frequently mentioned barriers to performing physical activity was being busy with other appointments followed by procrastination. On the other hand, participants found it easier to perform physical activity when it was part of their daily life. An example of how procrastinating resulted in not doing any physical activity anymore is as followed: *‘And then, I do it at the end of the day, I keep procrastinating... procrastinating... procrastinating...and eventually, at some point, the day is over and then I think yes… I will do it tomorrow’* (P1). One way to get physical activity in your daily routine is to have reminders. One participant had a smart solution to remind himself to perform some physical activity: *‘I also have a pull-up bar in my hall, so when I go to the toilet, I always see it. So that is a kind of a reminder of Oh yes, I still have to do something’* (P3). Reminders were an often-mentioned trick for participants to perform physical activity.

#### Self-compassion

Self-compassion is more mentioned among participants that were in ‘the active during both’ group. In addition, it was often mentioned as a wish, that it would help them to become more active in the other groups. An example of how self-compassion can work is described as followed: *‘By writing a positive diary. So, you can write down: I have not put any pressure on myself by obsessively exercising or umh, by having to do that workout, no I have at least moved and that’s fine. And I took care of myself, I listened to my body’* (P16).

#### Mental complaints/lack of energy/negative thoughts

Mental complaints (e.g. low mood, mental fatigue, anxiety) and a lack of energy were the most frequently mentioned barriers next to the weather. One participant explained when ‘*a mental breakdown*’ (P7) is experienced, it is more difficult to do something active. When people are tired, low in energy, or did not sleep well it was also difficult to perform some active activities. Another frequently mentioned barrier was having negative thoughts. One of the participants describes: *‘I also get negative thoughts like “Oh I cannot do this at all” and “Oh this is not going well” or “I am not in shape” and “I will never get better at this”*’ (P9). Another participant described how her thoughts are holding her back to perform physical activities *‘For me, it is mainly the way I approach it myself. Because I gave it such a negative connotation. Somewhere down the road, I made it an obligation for myself. So let me just say the mindset about it, that I would like to change for myself’* (P17).

#### Physical complaints/(fear of) injury

Physical complaints or injuries often caused inactivity among the participants. Some physical conditions experienced were cardiac infarction, vertigo (dizziness) and hypermobility of the joints. Other people had an injury (in the past) or were discouraged to perform a physical activity because of their low aerobic fitness. People that (had) experienced an injury, expressed a fear of a new injury. This was another reason to be hesitant in performing physical activity. An example of how a participant described the fear of an injury: ‘*But, yes, I would like to do some exercises, but on the other hand, I am like, I do not want to do something wrong that can cause pain in my leg again*’ (P5).

#### Goals as a method to overcome barriers

About half of the participants set goals for their daily and/or moderate/vigorous physical activities. Some of the participants had a general idea of what they wanted to achieve but did not set clear goals for themselves. Across all groups, it was mentioned that when they wanted to achieve their goal, it was helpful to become more active. In general, people with clear goals, perform more physical activity. Five participants recommended setting goals for other people that want to become more physically active. An overview of mentioned goals can be found in Appendix 6. All manuscripts and available data can be found on https://osf.io/3puer/.

## Discussion

This study provided new information about facilitators and barriers among people with moderate mental disorders to perform physical activity. First, most participants find physical activity important and experience a positive attitude towards it. However, not all of them have a sufficient level of physical activity and most expressed the intention to increase their physical activity level. The more confident people felt, the more physically active they were. A high social norm does not always have to be beneficial. However, almost everyone experienced enough positive social support, which was mentioned by all participants as a facilitator. Having fun was second-mentioned as a facilitator, with not having fun as a critical barrier. In addition, weather, performing the physical activity as part of a daily routine, self-compassion, and being in a nice environment were the most emphasized facilitators. Being busy, procrastinating, and having mental/physical complaints were the most mentioned barriers to becoming active. In general, these determinants are similar to the determinants among healthy people in previous studies (Biddle and Mutrie 2007; Choi et al. 2017; Giles-Corti and Donovan 2002). However, specific facilitating factors and barriers were emphasized in this study (e.g. self-compassion).

### Facilitating factors and barriers to physical activity

Most of the facilitators mentioned by the participants were related to social influences and their behavioural regulation. These results are similar to the scoping review of Glowacki et al. (2017). However, our study also found a unique facilitator among people with mental disorders, namely *self-compassion*. This facilitator was mentioned by participants that had a highly positive attitude towards physical activity. Participants mentioned that self-compassion helped them to accept the situation, to take some rest and go ahead the next day. Participants who experienced many negative thoughts also mentioned that it would help to have more self-compassion. So self-compassion can be helpful to increase physical activity; in addition, self-compassion also predicts mental well-being in general (Kotera et al. 2021). Our study shed light on the importance of self-compassion, which can be used for the development of future (online) interventions.

Previous research found that professional support and accessibility were important to start with physical activity among people with mental disorders (Rebar and Taylor 2017). However, our study showed that social support, a buddy, and commitment were more important than professional guidance. To continue physical activity it is important to have a meaningful experience (Rebar and Taylor 2017). This was explained by one participant who expressed the goal to become her more active self again. She suffered from a cardiac infarction and had to take medication that kept her energy low. Despite these barriers, she expressed motivation and willpower to maintain her physical activity so she could have a meaningful life to herself again. Giving a meaningful experience to their physical activity was found among participants that had clear goals. Among the group with clear goals, participants were maintaining their physical activity level for a longer period.

### Barriers to physical activity

Half of the frequently mentioned barriers are in the mood domain, which is in line with previous research (Glowacki et al. 2017; Rebar and Taylor 2017). Higher levels of mental disorders and lower levels of self-efficacy were found as barriers, which is similar to other studies (Searle et al. 2011; Vancampfort et al. 2015). Other factors we found that made it more difficult for participants to perform physical activity were being busy with other appointments, procrastination, the weather and lack of social support (Glowacki et al. 2017; Pelletier et al. 2017). However, costs were only mentioned once in our study, which is different from Glowacki et al. (2017) but similar to Pelletier et al. (2017). This difference could be because gyms were closed during the time of the interviews. Therefore, people did not have to incur costs to become active and other barriers were more prominent.

A frequently mentioned barrier in this study is physical complaints/(fear of) injury. This barrier is partly mentioned in other qualitative studies among people with mood- and/or anxiety-related disorders (Firth et al. 2016; Glowacki et al. 2017; Rebar and Taylor 2017). In previous studies mostly physical barriers were reported; however, this study also found fear of potential injuries as a barrier. This result could be because this sample experienced some injuries in the past that still influenced their physical activity level now. In sum, most of the mentioned barriers in the current study were found before; however, fear of an injury was more prominent in this population compared to other studies.

### Limitations and future research

Several limitations of this qualitative study should be noted. First, all the participants came from the same mental health institution which could jeopardize the diversity due to clinical settings. However, the participants were located all over The Netherlands and were not under treatment yet. Second, the interviewer used some leading questions, which can cause a bias in the participants’ answers. For example: ‘*How does staying in shape look like for you? Is that mainly aerobic fitness or physical appearance?*’ This question started open; however, then followed suggestions on what could have led participants in certain directions. To get a full range of answers, the interviewer asked several times about facilitators/barriers to give the participants space to talk freely. Third, not all detected overarching themes were discussed during all the interviews such as social norms or physical activity in the past. Some themes were not planned in the original set of questions, which caused some different discussed themes across interviews. This caused difficulties in the interpretation of the interviews. Additionally, the later conducted interviews were more detailed compared to the first few interviews. This is a logical result of the adjustments of the questions; however, it resulted in more specific beliefs, facilitators and barriers. Last, the interviewer gave compliments while participants talked about their physical activity experiences. This could jeopardize the neutrality of the interviews and people could have felt less comfortable talking about their physical inactivity. Despite these limitations, interviews were held until data saturation, and a full range of definitions and physical activity levels are found.

The results showed several determinants (attitude, social norms, self-efficacy, intention); however, the current study did not test a full theory as it was meant to discover new determinants, facilitators and barriers among people with moderate mental disorders. The current study focused not only on moderate/vigorous physical activity but also on daily movements. During the interview, this distinction is not always clear in the answers of the participants. Some difficulties arose during the interpretation process because the two topics were intertwined in the interviews. Future research could divide the interview topics into daily movements and moderate/vigorous physical activity. Specific beliefs, facilitators, and barriers could then be investigated within the same person but across the two physical activity groups. Another recommendation for future research is to focus more on the background of the participants to find out how specific attitudes and beliefs have originated. This new information can help us understand how and why some people have a strong positive attitude towards physical activity. Future research could focus more on the distinction between people with a strong positive attitude and a negative/neutral attitude. At least, future research should conduct a quantitative study to confirm our results and to say something about generalizability.

## Conclusion

In general, people with mental disorders experience a positive attitude towards physical activity, and they consider it important. This is similar to healthy participants from previous research. However, people still encounter some difficulties to become (more) active and showed the intention to increase their physical activity level. People with mental disorders encounter specific facilitators and barriers, compared to healthy controls, such as the newfound facilitator of self-compassion: ‘self-understanding and self-kindness aimed at easing suffering during times of hardness’ (Kotera et al. 2021). Other emphasized facilitators are social support, having fun during physical activity, noticing progress, making physical activity part of their routine, and being in a nice environment. The current study confirmed some common barriers such as being busy, a lack of energy, negative thoughts, procrastination, and mental- and physical complaints.

In conclusion, the following suggestions can be made for the development of an (online) intervention to increase physical activity among people with moderate mental disorders. Encourage people to find physical activities they enjoy. When they experience difficulties to being active alone, encourage them to find someone to commit to, also during times they feel low. Start a diary about the made progress and let people write down how they feel. Emphasize little steps and let them create reminders for themselves, so people can integrate physical activity into their daily/weekly life. When people experience negative thoughts or mental disorders, encourage them to be kind to themselves, let them listen to their bodies, and let them practice self-compassion. Let people look back at what they wrote down in their diary and soon they experience progress, which will motivate them again. A positive vicious cycle is round.

## Data Availability

Atlas.ti version 9.

## Appendix 1

Table 1

**Table 1:** COREQ guideline table

No	Item	Guide question/description
Domain 1: Research team and reflexivity		
*Personal characteristics*		
1.	Interviewer/facilitator	First author
2.	Credentials	Master student
3.	Occupation	Psychologist at institution of recruitment
4.	Gender	Female
5.	Experience and training	Fairly new to qualitative research (supervised by a senior researcher)
*Relationship with participants*		
6.	Relationship established	For half of the participants, it was the first time they saw the interviewer. The other half had seen the interviewer during the pre-intake or in a workshop. There was little time for establishing a relationship
7.	Participant knowledge of the interviewer?	Participants knew about reasons for doing the research
8.	Interviewer characteristics	Participants did not report characteristics of the interviewer. Self-reflections of the interviewer are written down in the discussion
Domain 2: Study design		
Theoretical framework		
9.	Methodoloigcal orientation and theory	Generic qualitative research approach
*Participants selection*		
10.	Sampling	Convenience sampling
11.	Method of approach	Telephone
12.	Sample size	Seventeen
13.	Non-participation	Eight Reasons: did not pick up the phone, not interested in the topic or treatment was already a lot of work
*Setting*		
14.	Setting of data collection	From home
15.	Presence of non-participants	No other people besides the participant and researcher were present
16.	Description of sample	Diagnosis is an important characteristic of the sample
*Data collection*		
17.	Interview guide	The main questions are attached in appendix 3. Interview questions were not pilot tested
18.	Repeat interviews	The main questions are attached in appendix 3. Interview questions were not pilot tested
19.	Audio visual recording	Three-quarters of the interviews were videotaped with only the interviewer visible. One-quarter of the interviews were audiotaped
20.	Fieldnotes	Notes were taken after the interviews
21.	Duration	30 minutes
22.	Data saturation	Yes, data saturation is discussed
23.	Transcripts returned	Yes, abstracts of transcripts were returned to participants for comments corrections
Domain 3: Analysis and findings		
*Data analysis*		
24.	Number of data coders	One main coder. Checked by senior researcher
25.	Description of the coding tree	No, a coding three is not attached to the paper
26.	Derivation of theses	Themes were mainly identified in advance
27.	Software	Atlas.ti version 9.0
28.	Participant checking	Yes, one person asked for corrections. The corrections were made
*Reporting*		
29.	Quotations presented	Yes, participants quotations are presented to illustrate themes with a participation number
30.	Data and findings consistent	Yes, there is consistency between the presented data and the findings
31.	Clarity of major themes	Yes, major themes were presented in the findings
32.	Clarity of minor themes	Partly, only a few minor themes were discussed in the findings

## Appendix 2

### Introduction text of invitation interview

*"Through this interview, we would like to examine what factors have a positive and what factors have a negative effect on our physical activity. Your information will be used to improve the online intervention that Oh My Mood is developing. In addition, your information will be used for further analysis. The interview will be about 30–45 minutes and we would like to ask your permission to audiotape the interview for transcription."*

## Appendix 3

### Example questions/topics for the interviews

1. How much physical activity do you perform in your daily life?
2. What is your definition of physical activity?
3. What do you think/is your opinion of physical activity?
4. How important is physical activity to you?
5. How satisfied, in general, are you with your physical activity level?
  - What do you need to become more satisfied?
  - What are motivational factors to you to become more physically active?
  - Or:
  - What makes you completely satisfied?
  - What makes it easy for you to perform physical activity?

6. Are there moments when it is difficult for you to perform physical activity?
7. What can you do yourself to make physical activity easier?
8. What can your surroundings do to perform more physical activity?
  - Do you get enough social support from people around you?
  - What kind of support? Is it working counterproductive?

9. Do you have the intention to perform more physical activity?
10. Would you like to get more support to become more physically active through an eHealth intervention?
11. What would you like to see back in this eHealth intervention?
12. What would you like to achieve with physical activity?
  - Do you currently set goals regarding physical activity?

## Appendix 4

Table 2

**Table 2:** Table with participants’ definition of physical activity

Definition	(N)
*Physical activity is physical, but also a movement for the mind*	1
*From standing up to more vigorous physical activity*	2
*Using staircase, walking, biking, active job, household tasks and vigorous physical activity*	4
*Performing physical activities with awareness such as walking, biking and vigorous movements*	6
*Physical activities with awareness, including being satisfied afterwards.*	1
*More vigorous physical activity and outdoor activities*	1
*Only vigorous physical activity, including sweating and being satisfied afterwards*	2

## Appendix 5

Table 3

**Table 3:** Overview of facilitators, barriers and tricks to become more physically active

Facilitator (N)	Barrier (N)	Tricks (N)
Social support/buddy/commitment (17)	Lack of social support/commitment (4)	Talking to others to find out own strengths (2)
		Solidarity to partner (1)
Having fun during physical activity (11)	Not having fun during physical activity (9)	Motivational music (2)
		Find a sport you are good at (1)
		Find a sport with competition (1)
Good weather (11)	Bad weather (11)	
Notice progress (11)		
Physical activity is part of daily routine (9)	Physical activity is not part of daily routine (4)	Fast start of physical activity (7)
	Busy with other appointments (11)	Reminders (7)
	Procrastination/sit on couch (7)	Planning (6)
	Stop of physical activity (4)	Convenient times of group classes (1)
	Different routine (3)	
Self-compassion (9)	Lack of self-compassion (3)	Acceptation (1)
	High demands on yourself (4)	Mindful walking/exercising (2)
	Physical complaints/(fear of) injury (7)	Find your new physical activity baseline (4)
	Medicines (1)	
Being in a nice environment (9)		
Want to achieve goals (8)	Unrealistic goals (4)	Taking little steps (5)
Internal motivation (7)		Motivational self-talk (4)
		Past successes (4)
		Internal checklist (2)
Mental Health (5)	Mental complaints (11)	Breaking vicious cycle (4)
	Lack of energy (11)	Seeking mental health care (1)
	Negative thoughts (6)	Listen to your body (4)
	Not feeling comfortable (2)	
Living close to nature (5)		Dog has to go for a walk (2)
Personal trainer/guidance (4)		
Active work (4)	Work/school from home (6)	Going back to work (2)
	Office work (4)	
Household tasks (3)	Household tasks (2)	
Get more background knowledge (3)		
External motivation (3)		Rewards (4)
Focus (3)		
Curious to different sports (2)		
Distance to physical activity (2)	Closure of gyms (6)	
	Smoking (2)	Increasing other health aspects such as eating (2)
Compliments from others (2)		
Resilience (1)		
Child that wants to play (1)	Young children at home (2)	Nanny for children (1)
Not having a car (1)	Having a car (1)	
Autonomy (1)	Lack of autonomy (1)	
	Menstrual cycle (1)	
	Financial costs (1)	

## Appendix 6

Table 4

**Table 4:** Participants’ goals

Participants	Goals
1	Clear goal: *‘At the moment I actually have the goal to burn 500 calories every day on an indoor exercise bike’.*
2	Clear goal: *‘Even if it will take me another five years to get there, I don’t care you know. It is my goal to be able to move normally again’.*
3	No goals: *‘I: Have you set certain goals for yourself now? P3: Umh, no. I: no, okay. Have you ever thought about that? P3’ Umh, no, not really.’*
4	Clear goal: *‘Umh, at the moment I actually want to apply for military training at the end of the year’.*
5	No goals: *‘I: Do you currently have goals? P5: Umh, yes and no. Because I have an injury in my left lower leg. And for that I am being treated at the physiotherapist’.*
6	No clear goals right now. If participant performs more moderate/vigorous physical activity clear goals are set. *‘I will set myself a goal, umh, well, in three months I want to do that 5-kilometre run’.*
7	No clear goals right now. Goals helped in the past. *‘I: Do you set a goal for yourself? P7: No, actually not’.*
8	Clear goals: *‘I say okay, I have to do ‘Nederland in beweging’, at least five times a week. On the weekend I can say, well not now’.*
9	No clear goals in general. On days off participants says *‘I want to do my work-out before the lunch’* But also *‘I: Do you set goals when you exercise? P9: Usually I start something and then I see what happens’.*
10	No clear goals: more general as precaution. *‘I don’t want to get older and eventually be unable to do anything because I never did some physical activity. So, you become very dependent and then you can hardly walk well or are not used to it and you get tired very quickly at a later age. I think I want to avoid that’.*
11	Clear goals for household tasks. *‘Sort of setting goals for myself, like “oh this week I’m going to do …”, start to plan again what I’m going to do this week’.*
12	Clear goals: *‘I basically try to go to the woods with my bike two or three times a week’.*
13	Clear goals: *‘Umh, Nah I would really like to start biking again’. A*nd also*: ‘Soon another hobby of mine starts, so that motivates me to work on my fitness level. So, I can be back on the filed in a decent way.’*
14	No clear goals: *‘Well, it is mainly that I do want to get a little fitter and I want to lose a little bit of weight’.*
15	No clear goals: *‘I: did you set goals for yourself in the past? P15: Yes. I: And did you feel that it helped somewhat? P15: No, no, no, only very briefly each time’.*
16	Clear goals: *‘My goal is to exercise intensive three times a week, as I did this morning. And next to that I want to walk at least half an hour every day outside’.*
17	Clear goals: *‘Yes, I have a route with walking, which I can do in several ways, but yes of which I always do the 40-minute route’.* But also: *‘Umh, but I do know that I find it more difficult to set goals with running than when I go for a walk’.*
